# Are gender-specific approaches needed in diagnosing early axial spondyloarthritis? Data from the SPondyloArthritis Caught Early cohort

**DOI:** 10.1186/s13075-018-1705-x

**Published:** 2018-10-01

**Authors:** Augusta Ortolan, Miranda van Lunteren, Sofia Ramiro, Roberta Ramonda, Robert B. M. Landewé, Hanne Dagfinrud, Lennart T. H. Jacobsson, Désirée van der Heijde, Floris A. van Gaalen

**Affiliations:** 1 Department of Rheumatology, Leiden University Medical Center, Leiden, the Netherlands; 2 Rheumatology Unit, Department of Medicine (DIMED), University of Padua, Padua, Italy; 3 Department of Rheumatology, Amsterdam Rheumatology and Immunology Center, Amsterdam, the Netherlands; 4 Department of Rheumatology, Zuyderland Medical Center, Heerlen, the Netherlands; 5 Department of Rheumatology, Diakonhjemmet Hospital, Oslo, Norway; 6 Department of Rheumatology, University of Gothenburg, Gothenburg, Sweden

**Keywords:** Spondyloarthritis, Gender, Diagnosis, Imaging

## Abstract

**Background:**

Although gender differences have been observed in the severity of axial spondyloarthritis (axSpA), gender differences in disease presentation of early axSpA have not been thoroughly investigated. In particular, their impact on the diagnostic process is unknown.

**Methods:**

Baseline data from the SPondyloArthritis Caught Early cohort, which includes patients with chronic back pain (CBP; duration ≥ 3 months and ≤ 2 years, age of onset < 45 years), were analysed. Patients underwent a full diagnostic work-up, including MRI and radiograph of the sacroiliac joints (MRI-SIJ and X-SIJ), to establish a diagnosis of axSpA. Characteristics of male and female patients with a certain diagnosis of axSpA (confidence level by the physician ≥ 7 on a 0–10 rating scale) were compared. Regression models were built for: the whole CBP cohort stratified by gender, to study which SpA features were associated most with diagnosis in each gender; and for axSpA patients, to test whether gender was associated with imaging positivity (MRI-SIJ^+^ and/or X-SIJ^+^).

**Results:**

Of the 719 CBP patients, 275 were male. With 146/275 males and 155/444 females diagnosed as axSpA, males were more likely to be diagnosed with axSpA (OR 2.1, 95% CI 1.5–2.9). Despite similar symptom duration, male axSpA patients were younger at diagnosis (27.4 ± 7.5 vs 29.5 ± 7.8 years; *p* = 0.02). Presence of SpA features was similar in male and female axSpA patients, except for HLA-B27 and imaging positivity that were more common in male axSpA patients (80% vs 60%; *p* < 0.01 and 78% vs 64%; *p* = 0.01). Nevertheless, these SpA features were still more prevalent in female axSpA patients than in no-axSpA patients, both females (HLA-B27^+^ 23%, positive imaging 7%) and males (HLAB27^+^ 34%, positive imaging 11%) (all *p* < 0.01). Moreover, in multivariable models with diagnosis of axSpA as outcome, HLA-B27 and imaging positivity were associated with the diagnosis in both sexes. In models with imaging positivity as outcome, male gender and HLA-B27 were both independently associated with MRI^+^ and/or X-SI^+^.

**Conclusions:**

While our data show clear gender differences in early axSpA, they highlight that HLA-B27 and imaging are still key elements for diagnosis in both genders. Our study does not suggest that separate diagnostic strategies for men and women are required.

**Electronic supplementary material:**

The online version of this article (10.1186/s13075-018-1705-x) contains supplementary material, which is available to authorized users.

## Background

Spondyloarthritis (SpA) refers to a group of chronic inflammatory diseases that, according to the Assessment of SpondyloArthritis international Society (ASAS) classification criteria, may be divided by the predominant site of involvement into axial SpA (axSpA) and peripheral SpA [1, 2]. In addition, axSpA can be further split into radiographic axSpA (r-axSpA, also known as ankylosing spondylitis), characterised by typical radiographic changes in sacroiliac joints (SIJ) [3], and non-radiographic axSpA, a form of the disease in which structural damage in the SIJ is not (yet) present.

Although axSpA affects both men and women, r-axSpA is more common in males, with a reported male to female ratio of 2–3:1 [4–6]. The higher incidence of r-axSpA in males compared to females has stimulated research on gender differences in this disease. In patients with longstanding r-axSpA, males have more severe radiographic damage and often more inflammation [7–11]. On the other hand, some authors have reported that female r-axSpA patients more often have cervical spine involvement, peripheral pain, and worse patient-reported outcomes (PROs) [7, 10, 12, 13]. Moreover, a longer time frame between symptom onset (based on patient history) and diagnosis has been repeatedly noticed in female r-axSpA patients [9, 14]. This finding poses the question of whether there is an earlier presentation of the disease in males or an earlier detection of the disease after its onset. If there were phenotypical gender differences at the start or diagnosis of the disease, with less typical findings in women, this could result in a delayed diagnosis. The importance of gender differences in the entire axSpA spectrum has only recently been appreciated, especially since an earlier diagnosis of axSpA has become more feasible. Few studies have been performed in cohorts with early disease such as the German Spondyloarthritis Inception Cohort (GESPIC) and the DEvenir des Spondylarthropathies Indifférenciées Récentes (DESIR) cohort [15, 16], with only one report specifically focusing on possible gender differences [16]. In the GESPIC cohort, composed of patients with a certain diagnosis of axSpA and a mean disease duration approaching 4 years, male gender was independently associated with more radiographic damage [15]. In the DESIR cohort, which includes patients with a high likelihood of SpA (inflammatory back pain highly suspected of axSpA), in the subgroup of patients who fulfilled the ASAS criteria, males were younger at diagnosis, had higher C-reactive protein (CRP) levels, and more often had radiographic sacroiliitis and/or signs of inflammation of SIJ and the spine on magnetic resonance imaging (MRI) [16]. Both studies include only patients with an established (or at least very likely) diagnosis and do not have subjects without axSpA for comparison.

Given the clear gender difference in established r-axSpA, it would be important to know whether differences between male and female axSpA patients are already present early in the disease. If so, different diagnostic strategies may be needed for males and females, considering that diagnostic delay is reported to be higher in females [17, 18]. Therefore, we set out to analyse gender differences at the moment of diagnosis of axSpA in a large inception cohort of chronic back pain (CBP) patients of unknown origin. Our aim was to assess differences in disease presentation between males and females in early axSpA, and to determine whether different SpA features are important for the diagnosis of axSpA in the two genders, possibly demanding separate diagnostic strategies.

## Methods

### Study design and population

The SPondyloArthritis Caught Early (SPACE) cohort is an ongoing prospective multicentre study which was initiated in January 2009 and has been described in detail elsewhere [19]. In brief, patients with CBP (≥ 3 months and ≤ 2 years of duration) of unknown origin and age of onset < 45 years are included. Patients have been recruited from six different rheumatology outpatient clinics in the Netherlands (Amsterdam, Gouda, Leiden), Norway (Oslo), Sweden (multiple sites), and Italy (Padua). For the present analysis, baseline data were available for 740 patients (SPACE database lock, March 2017). Patients with missing information on clinical diagnosis or imaging were excluded (*n* = 21), resulting in 719 patients who could be included in the current analyses.

Patients enrolled in the cohort underwent a full diagnostic work-up, including the assessment of SpA features according to the definitions of the ASAS criteria [2]. Consequently, the actual presence or history of the following SpA features was ascertained: inflammatory back pain (IBP), good response to non-steroidal anti-inflammatory drugs (NSAIDs), positive family history of SpA (family history in first or second-degree relatives of r-axSpA, uveitis, reactive arthritis, inflammatory bowel disease, or psoriasis), peripheral arthritis, dactylitis, enthesitis, acute anterior uveitis, inflammatory bowel disease, and psoriasis. Additional details from the patient history were recorded, including their smoking history which was treated as a categorical variable (yes/no/past smoker). The clinical examination included tender/swollen joint and the Maastricht Ankylosing Spondylitis Enthesitis Score (MASES) [20].

In addition to the clinical SpA features, CRP, the erythrocyte sedimentation rate (ESR), and human leukocyte antigen (HLA)-B27 were measured, and plain radiographs of the pelvis (X-SIJ) in the anteroposterior view and MRI-SIJ were performed. Each centre interpreted X-SIJ and MRI-SIJ with respect to the presence or absence of sacroiliitis using global assessment as part of routine clinical practice (local reading), with radiologists and rheumatologists whom had specifically been asked whether there was evidence of sacroiliitis.

Moreover, radiographs of the spine (X-spine) in the sagittal view were taken and centrally scored by three well-trained readers. A syndesmophyte was considered present if detected at the same vertebral corner by at least two readers.

Based on the collected local information, rheumatologists provided a diagnosis of ‘axSpA’ or ‘no axSpA’. In addition, they were requested to provide a level of confidence for their diagnosis on an 11-point numerical rating scale ranging from 0 (not confident at all) to 10 (very confident).

In this study, we considered as ‘axSpA patients’ those who received a diagnosis of axSpA by the rheumatologist with a level of confidence ≥ 7. This subgroup is hereafter simply referred to as axSpA. ‘No-axSpA patients’ included patients with a diagnosis of ‘no axSpA’ and patients with an uncertain diagnosis of axSpA (level of confidence < 7).

### Statistical analysis

Patient and disease characteristics were compared between male and female patients, in both groups of axSpA and no-axSpA patients, using the Mann–Whitney *U* test or *t* test for continuous variables and the chi-square or Fisher exact test, as appropriate. Results are presented as frequency (%) or mean (standard deviation (SD)). In order to evaluate which SpA features contributed most to the clinical diagnosis of axSpA, multivariable logistic regression models were built using the whole SPACE cohort stratified by gender. SpA features were used as the independent variables of interest, and a certain diagnosis of axSpA as the outcome. The SpA features were forced into the multivariable regression models, regardless of their significance in univariable analyses.

Furthermore, in axSpA patients, the relationship between gender and imaging abnormalities was investigated through logistic models with X-SIJ^+^, MRI-SIJ^+^ or any positive imaging (X-SIJ^+^ and/or MRI-SIJ^+^) as the outcomes. Independent variables, apart from gender, consisted of possible determinants of the outcome or potential confounders based on previous studies [21, 22], namely positive family history of SpA, elevated CRP or ESR, and variables that had shown a statistically significant difference between male and female axSpA patients in our baseline data. The independent variables showing an association with the outcome in univariable analyses with *p* < 0.10 were subsequently included in the multivariable model.

Results of regression analyses were expressed in terms of odds ratio (OR) and 95% confidence interval (95% CI).

Data analysis was performed using STATA SE version 14 (StataCorp, College Station, TX, USA) and *p* ≤ 0.05 was considered significant.

## Results

A total of 719 patients with CBP were analysed. Their mean age was 29.0 (SD 8.2) years, and the mean CBP duration was 13.2 (6.9) months. The majority of patients (*n* = 444, 62%) were females, with a male to female ratio of 1:1.6. The clinical, laboratory, and imaging characteristics of the CBP population are presented in Table 1.

**Table 1:** Baseline characteristics and SpA features of chronic back pain patients included in the SPACE cohort (*n* = 719)

Characteristic	Value
Male gender, *n* (%)	275 (38)
Age at CBP onset (years), mean (SD)	29.0 (8.0)
Duration of CBP (months), mean (SD)	13.2 (6.9)
Alternating buttock pain, *n* (%) (*N* = 454^a^)	225 (55)
IBP, *n* (%)	499 (70)
Response to NSAIDs, *n* (%)	316 (45)
Family history of SpA, *n* (%)	305 (43)
Peripheral arthritis, *n* (%)	111 (15)
Heel enthesitis, *n* (%)	142 (20)
Dactylitis, *n* (%)	39 (5)
Uveitis, *n* (%)	58 (8)
Psoriasis, *n* (%)	88 (12)
IBD, *n* (%)	48 (6)
HLA-B27^+^, *n* (%)	315 (44)
Elevated CRP/ESR, *n* (%)	217 (30)
MRI-SIJ^+^, *n* (%)	234 (32)
X-SIJ^+^, *n* (%)	91 (12)
Any positive imaging,^b^ *n* (%)	250 (34)

*SpA* spondyloarthritis, *SPACE* SPondyloArthritis Caught Early, *CBP* chronic back pain, *IBP* inflammatory back pain, *NSAID* non-steroidal anti-inflammatory drug, *IBD* inflammatory bowel disease, *HLA* human leukocyte antigen, *CRP* C-reactive protein, *ESR* erythrocyte sedimentation rate, *MRI-SIJ* magnetic resonance imaging of sacroiliac joints, *X-SIJ* plain radiograph of sacroiliac joints

^a^Missing data < 5% unless otherwise indicated

^b^MRI-SIJ^+^ and/or X-SIJ^+^

Out of the 719 CBP patients, 301 patients (42%) were diagnosed with axSpA by the rheumatologist with a level of confidence (LoC) ≥ 7 (axSpA patients). An additional 82 patients (35% males) out of 719 received a diagnosis of axSpA with a level of confidence in diagnosis < 7 (here included in the no-axSpA group).

Of the 275 male CBP patients, 146 (53%) received a certain diagnosis of axSpA, and of the 444 female CBP patients, 155 (35%) received a certain diagnosis of axSpA, showing that male patients with CBP were significantly more likely to be diagnosed with axSpA (OR 2.1, 95% CI 1.5–2.9). When the patients with a low certainty diagnosis of axSpA (LoC < 7) were removed from the no-SpA group and added to the axSpA group, the gender difference remained the same (OR 2.0, 95% CI 1.5–2.7).

In the axSpA group, the mean age was 28.5 (7.8) years and the mean CBP duration was 13.3 (7.0) months. The male to female ratio was 1:1.1, with 146 (48%) males and 155 (52%) females. Male axSpA patients were younger than female axSpA patients at the time of diagnosis (27.4 (7.5) years vs 29.5 (7.8) years, *p* = 0.02; Table 2).

**Table 2:** Characteristics of patients without (*n* = 418) and with (*n* = 301) certain diagnosis of axSpA stratified by gender

	No axSpA		*p* value	axSpA		*p* value
	Male (*N* = 129)	Female (*N* = 289)		Male (*N* = 146)	Female (*N* = 155)	
Age at CBP onset (years), mean (SD)	29.3 (8.6)	29.4 (8.2)	0.9	**27.4 (7.5)**	**29.5 (7.8)**	**0.02**
Duration of CBP (months), mean (SD)	12.8 (6.9)	13.4 (7.0)	0.5	13.3 (7.1)	13.4 (7.0)	0.5
Alternating buttock pain, *n* (%) (*N* = 454^a^)	**23 (43)**	**100 (59)**	**0.03**	65 (60)	64 (51)	0.2
IBP, *n* (%)	83 (57)	164 (64)	0.2	123 (84)	129 (84)	1.0
Response to NSAIDs, *n* (%)	45 (37)	81 (29)	0.1	92 (64)	98 (66)	0.8
Family history of SpA, *n* (%)	44 (34)	124 (44)	0.1	68 (46)	69 (45)	0.8
Peripheral arthritis, *n* (%)	15 (12)	25 (9)	0.3	37 (25)	34 (22)	0.5
Heel enthesitis, *n* (%)	17 (13)	28 (10)	0.3	46 (31)	51 (33)	0.7
Dactylitis, *n* (%)	3 (2)	4 (1)	0.5	16 (11)	16 (10)	0.9
Uveitis, *n* (%)	7 (5)	12 (4)	0.6	19 (13)	20 (13)	1.0
Psoriasis, *n* (%)	6 (5)	23 (8)	0.2	22 (15)	37 (24)	0.051
IBD, *n* (%)	11 (8)	15 (5)	0.2	10 (7)	12 (8)	0.8
HLA-B27^+^, *n* (%)	**43 (34)**	**66 (23)**	**0.02**	**114 (80)**	**92 (60)**	**< 0.001**
Elevated CRP/ESR, *n* (%)	21 (16)	61 (21)	0.2	71 (49)	64 (42)	0.2
SpA features without imaging or HLA-B27, mean (SD)	2.4 (0.1)	2.1 (0.1)	0.3	3.5 (1.7)	3.5 (1.6)	1.0
MRI-SIJ^+^/X-SIJ^–^, *n* (%)	7 (5)	19 (6)	0.6	65 (44)	68 (44)	0.9
MRI-SIJ^−^/X-SIJ^+^, *n* (%)	2 (1)	4 (1)	0.9	5 (3)	5 (3)	0.9
MRI-SIJ^+^/X-SIJ^+^, *n* (%)	**5 (4)**	**0 (0)**	**0.01**	**44 (30)**	**26 (17)**	**0.006**
Any positive imaging,^b^ *n* (%)	14 (10)	23 (7)	0.3	**114 (78)**	**99 (64)**	**0.007**
Number of syndesmophytes, mean (SD) (*N* = 182^a^)	n/a	n/a	–	0.1 (0.4)	0.0 (0.1)	0.5
Patients with syndesmophytes, *n* (%) (*N* = 182^a^)	n/a	n/a	–	**7/83 (8)**	**1/99 (1)**	**0.02**
Current smokers, *n* (%)	33 (28)	54 (19)	**0.1**	**31 (22)**	**15 (10)**	**0.02**

Modified Stoke ankylosing spondylitis Spine Score available for 182/301 axSpA patients. Bold data indicate significant results

*axSpA* axial spondyloarthritis, *CBP* chronic back pain, *IBP* inflammatory back pain, *NSAID* non-steroidal anti-inflammatory drug, *SpA* spondyloarthritis, *IBD* inflammatory bowel disease, *HLA* human leukocyte antigen, *CRP* C-reactive protein, *ESR* erythrocyte sedimentation rate, *MRI-SIJ* magnetic resonance imaging of sacroiliac joints, *X-SIJ* plain radiograph of of sacroiliac joints

^a^Missing data < 5% unless otherwise indicated

^b^MRI-SIJ ^+^ and/or X-SIJ^+^

Among axSpA patients, most SpA features were similarly distributed between the two genders. However, males were significantly more often HLA-B27^+^ (80% vs 60%; *p* < 0.001) and more often had imaging abnormalities (any positive imaging 78% vs 64%; *p* = 0.007) than axSpA female patients. The latter was mainly due to the fact that more males than females had both a positive MRI-SI and a positive X-SI (30% vs 17%; *p* = 0.006). Moreover, although syndesmophytes were uncommon in these patients with early disease, more males than females had syndesmophytes present (8% vs 1%; *p* = 0.02). In contrast, psoriasis appeared to be more common in females (24% vs 15%), although this difference was not statistically significant (*p* = 0.051). Interestingly, the total number of SpA features without HLA-B27 and imaging was not different between males and females (3.5 (1.7) vs 3.5(1.6); *p* = 0.99). Females were less frequently smokers than males (10% vs 22%; *p* = 0.02).

In both genders, all SpA features were significantly more common in axSpA patients as compared to no-axSpA patients except for IBD and family history. The latter was actually more common in male axSpA patients compared to male no-axSpA patients, but such a difference was not observed in female patients. Interestingly, HLA-B27 and positive imaging were still more prevalent in female axSpA patients than in no-axSpA patients, both females (HLA-B27^+^ 23% and positive imaging 7%) and males (HLAB27^+^ 34% and positive imaging 11%) (all *p* < 0.01).

The multivariable regression model in male CBP patients, with a certain diagnosis of axSpA as an outcome, showed that HLA-B27 positivity, elevated CRP and/or ESR, and MRI-SIJ^+^ were independently associated with the diagnosis of axSpA (Table 3).

**Table 3:** Multivariable model with certain diagnosis of axSpA (level of confidence ≥ 7/10) as an outcome and SpA features as independent variables in male CBP population

Independent variable	OR (95% CI) (*N* = 262^a^)	*p* value
HLA-B27^+^	**3.8 (1.7–8.8)**	**< 0.001**
IBP	2.1 (0.8–5.6)	0.1
Uveitis	1.7 (0.5–6.0)	0.3
Psoriasis	2.7 (0.6–11.2)	0.2
Heel enthesitis	2.3 (0.8–6.5)	0.09
Dactylitis	0.3 (0.0–2.4)	0.3
IBD	1.8 (0.4–7.6)	0.4
Peripheral arthritis	1.9 (0.6–6.0)	0.3
Response to NSAIDs	1.5 (0.6–3.3)	0.4
Family history of SpA	1.5 (0.7–3.6)	0.3
Elevated CRP/ESR	**2.5 (1.0–6.1)**	**0.05**
MRI-SIJ^+^	**24.3 (9.7–60.6)**	**< 0.001**
X-SIJ^+^	2.7 (0.7–9.4)	0.1

Bold data indicate significant results

*axSpA* axial spondyloarthritis, *SpA* spondyloarthritis, *CBP* chronic back pain, *OR* odds ratio, *CI* confidence interval, *HLA* human leukocyte antigen, *IBP* inflammatory back pain, *IBD* inflammatory bowel disease, *NSAID* non-steroidal anti-inflammatory drug, *CRP* C-reactive protein, *ESR* erythrocyte sedimentation rate, *MRI-SIJ* magnetic resonance imaging of sacroiliac joints, *X-SIJ* plain radiograph of of sacroiliac joints

^a^Number of patients with complete data on all variables

In the female CBP population, HLA-B27 positivity, MRI-SIJ^+^, and X-SIJ^+^ were independently associated with the diagnosis of axSpA in the multivariable model, but also other SpA features emerged as independent predictors of diagnosis; specifically a positive history or current presence of inflammatory back pain, uveitis, psoriasis, heel enthesitis, dactylitis, IBD, and response to NSAIDs (Table 4).

**Table 4:** Multivariable model with certain diagnosis of axSpA (level of confidence ≥ 7/10) as an outcome and SpA features as independent variables in female CBP population

Independent variable	OR (95% CI) (*N* = 424^a^)	*p* value
HLA-B27^+^	**6.7 (3.2–14.0)**	**< 0.001**
IBP	**4.0 (1.7—8.9)**	**0.001**
Uveitis	**3.1 (1.0–9.2)**	**0.04**
Psoriasis	**4.6 (1.8–12.0)**	**0.002**
Heel enthesitis	**4.3 (1.9–9.7)**	**< 0.001**
Dactylitis	**5.2 (1.0–26.9)**	**0.05**
IBD	**4.7 (1.5–14.8)**	**0.009**
Peripheral arthritis	1.4 (0.5–3.7)	0.5
Response to NSAIDs	**3.1 (1.6–6.0)**	**0.001**
Family history of SpA	0.9 (0.4–1.8)	0.8
Elevated CRP/ESR	1.7 (0.8–3.5)	0.2
MRI-SIJ^+^	**32.6 (14.2–75.0)**	**< 0.001**
X-SIJ^+^	**6.9 (1.4–32.7)**	**0.02**

Bold data indicate significant results

*axSpA* axial spondyloarthritis, *SpA* spondyloarthritis, *CBP* chronic back pain, *OR* odds ratio, *CI* confidence interval, *HLA* human leukocyte antigen, *IBP* inflammatory back pain, *IBD* inflammatory bowel disease, *NSAID* non-steroidal anti-inflammatory drug, *CRP* C-reactive protein, *ESR* erythrocyte sedimentation rate, *MRI-SIJ* magnetic resonance imaging of sacroiliac joints, *X-SIJ* plain radiograph of of sacroiliac joints

^a^Number of patients with complete data on all variables

In axSpA patients, the relationship between gender and imaging abnormalities was investigated. In the univariable regression analysis with positive imaging (MRI-SIJ^+^ and/or X-SIJ^+^) as the outcome (Table 5), an independent positive association (*p* < 0.05) was found for male gender, lower age at CBP onset, and HLA-B27 positivity. In the multivariable model, male gender and HLA-B27 positivity were independently and positively associated with imaging positivity. A positive family history of SpA showed a negative association in both the univariable analysis and the multivariable model.

**Table 5:** Factors associated with positive imaging (MRI-SIJ+ and/or X-SIJ+) in patients with certain diagnosis of axial spondyloarthritis (level of confidence ≥ 7/10)

Independent variables	Univariable analysis *N* = 289^a^		Multivariable model *N* = 289^a^	
	OR (95% CI)	*p*	OR (95% CI)	*p*
Male gender	**2.0 (1.2, 3.3)**	**0.007**	**1.8 (1.0, 3.1)**	**0.05**
Age at CBP onset	**1.0 (0.9, 1.0)**	**0.04**	1.0 (0.9, 1.0)	0.052
HLA-B27+	**2.0 (1.2, 3.4)**	**0.01**	**1.8 (1.0, 3.3)**	**0.04**
Positive family history of SpA^b^	**0.4 (0.3, 0.7)**	**0.002**	**0.4 (0.2, 0.7)**	**0.001**
Elevated CRP/ESR	1.6 (1.0, 2.7)	0.06	1.5 (0.9, 2.7)	0.1
Smoking (N=284^c^)	1.1 (0.8, 1.6)	0.4	NI	-

Data presented as odds ratio (95% confidence interval). Bold data indicate significant results

*MRI-SIJ* magnetic resonance imaging of sacroiliac joints, *X-SIJ* plain radiograph of of sacroiliac joints, *CBP* chronic back pain, *HLA* human leukocyte antigen, *SpA* spondyloarthritis, *CRP* C-reactive protein, *ESR* erythrocyte sedimentation rate, *NI* not included in multivariable model because *p* > 0.1 at univariate analysis and no confounder

^a^Number of patients with complete data on all variables

^b^Family history of spondyloarthritis according to the Assessment of SpondyloArthritis international Society includes family history in first or second-degree relatives of ankylosing spondylitis, uveitis, reactive arthritis, inflammatory bowel disease, or psoriasis

^c^Smoking data unavailable in five additional patients

Male gender was also positively associated with MRI-SIJ^+^ alone (OR 1.6, 95% CI 1.0–2.8) and with X-SIJ^+^ alone (OR 1.9, 95% CI 1.1–3.3) in the multivariable models having MRI-SIJ^+^ and X-SIJ^+^ separately as outcomes (data not shown).

Patient-reported outcomes, metrological indices, and disease activity indices of axSpA patients were recorded in the cohort and are presented in Additional file 1: Table S1. Bath Ankylosing Spondylitis Disease Activity Index (BASDAI) scores as well as the percentage of patients presenting with BASDAI > 4 were higher in females (4.5 (2.2) vs 3.4 (1.9) and 57% vs 34%; both *p* < 0.001). The Bath Ankylosing Spondylitis Functional Index (BASFI) and Short Form (SF)-36 outcomes were not significantly different between genders. Disease activity based on the Ankylosing Spondylitis Disease Activity Score (ASDAS) was similar in terms of absolute value, but females more frequently had a high or very high disease activity (ASDAS ≥ 2.1, 65% vs 46%; *p* = 0.004). Similarly, total MASES was similar between males and females, but the percentage of patients with current entheseal involvement (MASES > 0) was higher in females (70% vs 41%; *p* < 0.001). Finally, more female than male axSpA patients (43% vs 20%; *p* = 0.002) had an extreme BASDAI score (score ≥ 7 on a 0–10 scale in at least three questions of the BASDAI).

## Discussion

The analysis of gender differences in this large CBP cohort with short duration of complaints allowed us to conclude that, while females are the majority of patients in the original CBP population, males were twice as likely to be diagnosed with axSpA. At the moment of diagnosis of axSpA, males were younger and more often presented with HLA-B27 positivity and imaging abnormalities, including a higher prevalence of syndesmophytes, even though symptom duration was similar to female patients. However, the prevalence of most other SpA features was equally distributed between genders.

Although we noticed differences in disease presentation, the results of our study suggest that similar elements are relevant for the diagnosis of male and female axSpA patients. In fact, despite HLA-B27 positivity and imaging abnormalities being more common in male axSpA patients, they were significantly associated with diagnosing axSpA in both genders. We believe this is this first study to examine the possible relevance of different SpA features in making a diagnosis stratified for gender. This is the first report showing in a CBP cohort that males have a higher probability to be diagnosed with axSpA. Since all patients, suspected of axSpA or not, in our cohort underwent the same protocol (including extensive imaging), the gender difference cannot be attributed to differences in the diagnostic process.

Nevertheless, it is obvious that early axSpA does not have such a clear male gender skewing as r-axSpA, since female patients still represent nearly half of the axSpA population, as previously noted [15, 16]. This suggests that specific gender disparities (e.g. radiographic evidence of sacroiliitis) may become more apparent later in the disease course.

Our finding of male patients being younger at disease onset is in line with findings in the DESIR cohort [16]; considering the strict age span allowed by the inclusion criteria and given that males and females in our cohort had similarly short symptom duration, this likely reflects a true difference in the disease onset, rather than a better recognition of the disease in males. Previous reports on age of onset in male and female axSpA patients mainly concern longstanding disease, and some of them reported that males seemed to have an earlier onset [12, 23, 24]. The mechanism behind this is unclear and deserves further investigation.

The results of this study also confirm that imaging abnormalities are more frequently found in male axSpA patients, already at the beginning of the disease [15, 16, 24]. Moreover, we show that male sex is independently associated with positive imaging, both in terms of MRI inflammation and radiographic sacroiliitis. Remarkably, syndesmophytes were more common in males at baseline, showing that differences in radiographic damage between genders, which have been repeatedly observed in r-axSpA [7, 10, 11], can already be found in very early disease. Also, radiographic or MRI sacroiliitis was the most important item for diagnosis in women, and was significantly more common in both male and female axSpA patients compared to no-axSpA patients.

HLA-B27 was also more common in male axSpA patients, and the overall prevalence of HLA-B27 in axSpA patients in our study was similar to other early axSpA cohorts [25]. However, it has to be noted that in other axSpA populations, such as those of the clinical trials, where axSpA patients are included only if they fulfil ASAS criteria and not based solely on the rheumatologist diagnosis, often display higher percentages of HLA-B27^+^ patients [26, 27].

In studying the factors associated with sacroiliitis in patients with axSpA, a positive family history was protective of positive imaging even in univariate analysis. At present we have no clear explanation for this observation. Little is known about differences in axial spondyloarthritis between patients with and without a familial history. One study reported that familial ankylosing spondylitis was significantly milder than sporadic disease [28], while another found no difference in disease phenotype [29].

Although PROs were not the main outcome of our study, it is interesting to observe how, in line with data from the literature, female patients in our cohorts presented with a higher subjective disease burden [7, 10, 12, 13]. Unfortunately, our cohort lacks assessment of fibromyalgia, but the observation of female axSpA patients presenting with more extreme PROs than males suggests that fibromyalgia could have been a frequent co-morbidity for our axSpA patients, possibly influencing the degree of symptom complaint in females. Indeed, it has been described that about a third of axSpA patients could have a neuropathic pain component [30].

A clear limitation in our analysis is that our diagnostic models for axSpA do not truly reflect a normal diagnostic work-up in clinical practice, in which looking for—and excluding—other causes of CBP is pivotal. However, the validity of our observations derives from the fact that all of the information included in the model was available to the rheumatologist and was used at the moment of making the diagnosis (axSpA/no axSpA). On the other hand, rheumatologists certainly performed a diagnosis according to their background knowledge and beliefs, and therefore there could have been a tendency towards a specific interpretation of the results (e.g. women could have been under-diagnosed if the rheumatologists interpreted axSpA as a predominantly male disease). Finally, we cannot exclude that possible referral bias may have interfered with the proportion of males and females in the study population or with other results such as the nearly significant prevalence of psoriasis in females. Indeed, the reasons why GPs or medical specialists referred CBP patients to the rheumatologists is unknown, but they could have been more prone to refer a female CBP patient if SpA features (besides HLA-B27 and imaging) were already present or because they had worse symptoms, thus a channelling mechanism cannot be excluded. However, other cohorts have reported a higher frequency of CBP in females [31]; moreover, the male to female ratio in our axSpA population is in line with previous findings [15, 16].

## Conclusions

In summary, in patients with CBP of unknown origin, males are more likely to be diagnosed with axSpA. In addition, significant differences between genders, including age at onset and imaging abnormalities, already exist at a very early disease stage. Nevertheless, HLA-B27 and imaging are still key elements for a diagnosis of axSpA in both genders, and therefore our data do not support the need for gender-specific diagnostic strategies for early axSpA patients.

## Additional file

Additional file 1: **Table S1.** Patient-reported outcomes, metrological indexes, and disease activity indexes of patients with a certain diagnosis of axial spondyloarthritis (level of confidence ≥ 7), stratified by gender (*n* = 301) (DOCX 15 kb)

## Abbreviations

AS: Ankylosing spondylitis
ASAS: Assessment of SpondyloArthritis international Society
ASDAS: Ankylosing Spondylitis Disease Activity Score
axSpA: Axial spondyloarthritis
BASDAI: Bath Ankylosing Spondylitis Disease Activity Index
BASFI: Bath Ankylosing Spondylitis Functional Index
CBP: Chronic back pain
CRP: C-reactive protein
DESIR: DEvenir des Spondylarthropathies Indifférenciées Récentes
ESR: Erythrocyte sedimentation rate
GESPIC: German Spondyloarthritis Inception Cohort
HLA: Human leukocyte antigen
IBP: Inflammatory back pain
MASES: Maastricht Ankylosing Spondylitis Enthesitis Score
MRI: Magnetic resonance imaging
NSAID: Non-steroidal anti-inflammatory drug
PRO: Patient-reported outcome
SF-36: Short Form-36
SIJ: Sacroiliac joints
SpA: Spondyloarthritis
SPACE: SPondyloArthritis Caught Early
X-SIJ: X-rays of sacroiliac joints
X-spine: X-rays of the spine
