# Supplementary material for: Are gender-specific approaches needed in diagnosing early axial spondyloarthritis? Data from the SPondyloArthritis Caught Early cohort
Source: Arthritis Res Ther. 2018 Oct 1;20:218. doi: 10.1186/s13075-018-1705-x (PMC6167860; doi:10.1186/s13075-018-1705-x)
Supplement: Supplementary file 1 — Table S1. Patient-reported outcomes, metrological indexes, and disease activity indexes of patients with a certain diagnosis of axial spondyloarthritis (level of confidence ≥ 7), stratified by gender (n = 301) (DOCX 15 kb) [file 13075_2018_1705_MOESM1_ESM.docx]

**Table S1.** Patient reported outcomes, metrological indexes and disease activity indexes of patients with a certain diagnosis of axial spondyloarthritis (level of confidence≥7), stratified by gender (n=301)

|  | **Male**  **N=146** | **Female**  **N=155** | **p-value** |
| --- | --- | --- | --- |
| **CRP level (mg/L), mean (SD)** ***(N=293)** | 6.8 (9.7) | 6.9 (8.6) | 0.5 |
| **BASDAI (0-10), mean (SD) *(N=282)** | **3.4 (1.9)** | **4.5 (2.2)** | **<0.001** |
| **BASDAI >4, n (%) *(N=282)** | **47 (34)** | **83 (57)** | **<0.001** |
| **BASFI (0-10) , mean (SD) *(N=280)** | 2.0 (2.0) | 2.7 (2.4) | 0.4 |
| **BASFI >4, n (%) *(N=280)** | 27 (20) | 38 (26) | 0.2 |
| **SF-36 physical component summary (0-100), mean (SD) *(N=281)** | 28.2 (16.8) | 29.8 (13.0) | 0.4 |
| **SF-36 mental component summary (0-100), mean (SD) *(N=281)** | 46.5 (14.5) | 45.5 (12.6) | 0.5 |
| **ASDAS, mean (SD) *(N=280)** | 2.1 (0.9) | 2.3 (0.8) | 0.4 |
| **ASDAS ≥2.1, n (%)*(N=280)** | **63 (46)** | **90 (65)** | **0.004** |
| **MASES (0-13), mean (SD) *(N=215)** | 1.3 (2.2) | 3.0 (3.0) | 0.3 |
| **MASES>0, n (%)*(N=215)** | **53 (41)** | **97 (70)** | **<0.001** |

CRP=C reactive protein; BASDAI= Bath Ankylosing Spondylitis Disease Activity Index; BASFI= Bath Ankylosing Spondylitis Functional Index; BASMI= Bath Ankylosing Spondylitis Functional Index; ASDAS= Ankylosing Spondylitis Disease Activity Score using CRP; MASES=Maastricht Ankylosing Spondylitis Enthesitis Score; SF-36=short form-36. Bold indicates significant results.*Data available for the number of patients indicated in brackets
